# Prediction model for survival outcome in patients with acute myeloid leukemia: a multicenter prospective cohort study from Thai Acute Leukemia Working Group

**DOI:** 10.3389/fonc.2026.1901725

**Published:** 2026-08-26

**Authors:** Pirun Saelue, Jakrawadee Julamanee, Adisak Tantiworawit, Thanawat Rattanathammethee, Weerapat Owattanapanich, Smith Kungwankiattichai, Chantiya Chanswangphuwana, Chantana Polprasert, Wasithep Limvorapitak, Supawee Saengboon, Kannadit Prayongratana, Chantrapa Sriswasdi, Pimjai Niparuck, Teeraya Puavilai, Chajchawan Nakhakes, Chinadol Wanitpongpun

**Affiliations:** 1 Hematology Unit, Division of Internal Medicine, Faculty of Medicine, Prince of Songkla University, Songkhla, Thailand; 2 Division of Hematology, Department of Internal Medicine, Faculty of Medicine, Chiang Mai University, Chiang Mai, Thailand; 3 Division of Hematology, Department of Internal Medicine, Faculty of Medicine, Siriraj Hospital, Mahidol University, Bangkok, Thailand; 4 Division of Hematology, Department of Medicine, Faculty of Medicine, Chulalongkorn University and King Chulalongkorn Memorial Hospital, Bangkok, Thailand; 5 Research Unit in Translational Hematology, Chulalongkorn University, Bangkok, Thailand; 6 Division of Hematology, Department of Internal Medicine, Faculty of Medicine, Thammasat University, Pathumthani, Thailand; 7 Department of Internal Medicine, Phramongkutklao Hospital and College of Medicine, Bangkok, Thailand; 8 Division of Hematology, Department of Medicine, Ramathibodi Hospital, Mahidol University, Bangkok, Thailand; 9 Division of Hematology, Department of Medicine, Rajavithi Hospital, Bangkok, Thailand; 10 Hematology Unit, Department of Internal Medicine, Srinagarind Hospital, Khon Kaen University, Khon Kaen, Thailand

**Keywords:** acute myeloid leukemia, AML, prediction model, TALWG, THAI-LEDGE

## Abstract

**Introduction:**

Despite the development of advanced therapeutic approaches in the last two decades, acute myeloid leukemia (AML) has a poor prognosis, especially in older patients. The main causes of death are refractory/relapsed disease, fatal bleeding, or serious infection. A model to predict survival in patients with AML is necessary for clinicians to make decisions regarding appropriate treatment. In this study, we aimed to evaluate the predictive factors for death and generate a model to predict survival in patients with AML.

**Methods:**

We conducted a multicenter prospective cohort study across nine tertiary medical care institutes in Thailand, enrolling patients aged ≥ 18 years with newly diagnosed AML between January 1, 2014, and December 31, 2023. Patients with acute promyelocytic leukemia were excluded. Multivariable Cox proportional hazards regression analyses identified the predictors of mortality, and the final model was constructed using backward stepwise regression with Akaike Information Criterion selection. Model performance was assessed using Harrell’s C-index and calibration plots, with internal validation performed via bootstrapping.

**Results:**

A total of 1,055 patients were included. The median overall survival was 10.9 months, with a 10-year survival rate of 22.4%. Eight variables were independently associated with survival outcomes: age > 55 years, Eastern Cooperative Oncology Group performance status, tumor lysis syndrome, leukostasis, disseminated intravascular coagulation, white blood cell count, genetic risk, and type of induction therapy. The THAI-LEDGE model demonstrated a good discriminatory ability (C-index = 0.743) and satisfactory calibration. The internal validation yielded a C-index of 0.737, confirming the robustness of the model.

**Conclusions:**

The THAI-LEDGE model performed well in predicting the survival of AML patients. However, external validation of the model in other ethnic populations and healthcare settings are necessary before widespread clinical implementation.

## 1 Introduction

Acute myeloid leukemia (AML) is a heterogeneous group of myeloid neoplasms characterized by the proliferation of clonal immature myeloid cells in the bone marrow (1). Clinically, AML manifests as rapid bone marrow failure, including neutropenia, anemia, and thrombocytopenia (2). Induction chemotherapy with cytarabine for 7 days and anthracyclines for 3 days (7 + 3 regimen) is the cornerstone of AML treatment. This regimen is associated with improved overall survival (3). Hypomethylating agents with or without venetoclax serve as alternative treatments for older or unfit patients (4, 5). Approximately 60–70% of patients achieve complete remission after induction therapy. However, only 30% of patients achieve durable remission, resulting in long-term survival (6). Infection, bleeding, and refractory/relapsed AML are the main causes of death in patients with AML.

Among patients with AML, survival depends on multiple risk factors, including clinical and genetic factors. Advanced age is a crucial risk factor because it reflects patient’s tolerance to intensive chemotherapy or allogeneic stem cell transplantation (AlloSCT), particularly in patients with frailty or those aged >80 years (7, 8). Hyperleukocytosis, defined as a white blood cell (WBC) count >100,000 cells/µL at diagnosis, is a strongly poor predictive factor in these patients (9). In terms of genetic factors, *NPM1*-mutated AML without *FLT3-ITD* mutations or biallelic *CEBPA* mutations is a good predictive factor, whereas complex karyotypes or *TP53* mutations are poor predictive factors for survival in patients with AML (10).

Despite advancements in therapeutic approaches, including targeted therapy, antimicrobial prophylaxis, and supportive care, the prognosis of AML remains suboptimal, particularly in older patients, in contrast to the favorable prognosis of acute promyelocytic leukemia (APL) (7, 8, 11, 12). A prediction model could individually predict the survival of these patients, which would help clinicians make treatment decisions and plan future care. However, the models for predicting survival in patients with non-APL AML are limited. Thus, in the current study, we aimed to evaluate the predictive factors for death in patients with non-APL AML and generate a prediction model to predict the probability of survival outcomes in these patients.

## 2 Methods

### 2.1 Study design and population

This multicenter prospective observational study was conducted using the Thai Acute Leukemia Working Group (TALWG) database, which encompasses nine tertiary hospitals across Thailand. All adults (≥18 years) diagnosed with AML according to the 2008 or 2016 World Health Organization (WHO) diagnostic criteria (2, 13) who were included in a web-based registration between January 1, 2014, and December 31, 2023, including referral patients and patients treated at our own centers, were deemed eligible. Patients who lacked conventional genetic studies to classify genetic risk or were diagnosed with APL were excluded. This study analyzed data extracted from the TALWG registry database, for which approval was granted by the Ethics Committee for Research in Human Subjects at each study site; the original study was conducted in accordance with the Declaration of Helsinki. No sample size calculation was performed. All participants received standard treatment without any special intervention or incentives.

### 2.2 Predictors and outcomes

Variables collected during diagnosis included demographic, clinical, and laboratory parameters. Genetic risk was categorized according to the European LeukemiaNet 2022 classification (14). For the induction treatment of AML, the 7 + 3 regimen consisted of 7 days of cytarabine and 3 days of anthracyclines. Hypomethylating agents, including azacitidine/decitabine, with or without venetoclax, served as alternative treatments for patients who were unsuitable for the 7 + 3 regimen. Patients in the intermediate- or adverse-risk group were consolidated with AlloSCT or three to four cycles of high-dose cytarabine, depending on patient status or donor availability. Favorable-risk patients were consolidated by 3–4 cycles of high-dose cytarabine. The primary outcome was overall survival, defined as the time from diagnosis to death or the last follow-up.

### 2.3 Statistical analyses

Study results were analyzed using R program version 4.2.3 (R Foundation for Statistical Computing, Vienna, Austria). Baseline characteristics are presented as numbers with percentages for categorical data and medians with interquartile ranges (IQRs) for continuous data. A maximally selected log-rank statistic was used to determine the continuous variable cutoffs. We used the Kaplan–Meier method for overall survival analysis. Univariable and multivariable Cox proportional hazards regression analyses were performed to evaluate predictive factors for death in patients with AML. Hazard ratios were calculated for all variables in the univariable analysis. Variables with a P-value < 0.05 in univariable analysis were included in the multivariable analysis. All P-values were two-sided, and values of <0.05 were considered statistically significant. A sensitivity analysis was performed using continuous predictors in their original scale rather than categorized variables.

Before developing the prediction model, missing data were handled using regression imputation. Percentage of bone marrow blast values were imputed for 16 patients with missing data, and the imputed dataset was subsequently used for model development. All significant variables in the univariable analysis were analyzed using backward stepwise regression. The prediction model with the lowest Akaike Information Criterion (AIC) was selected as the best model for predicting the probability of survival. The proportional hazards assumption was evaluated using Schoenfeld residuals plot for each predictor included in the final Cox model and for the model overall. A sensitivity analysis was performed in which the prediction model was developed using LASSO-penalized Cox regression. The prediction model was presented as a nomogram to predict 1-month, 6-month, and 1-year survival rates in patients with AML. We evaluated model performance using Harrell’s C-index (C-index) and calibration plots. The prediction model was internally validated using a bootstrapping method with 500 resamples, which is the most efficient method for internal validation studies (15). The C-index and calibration plots were used to internally validate the model.

## 3 Results

### 3.1 Baseline characteristics

**Figure 1:**
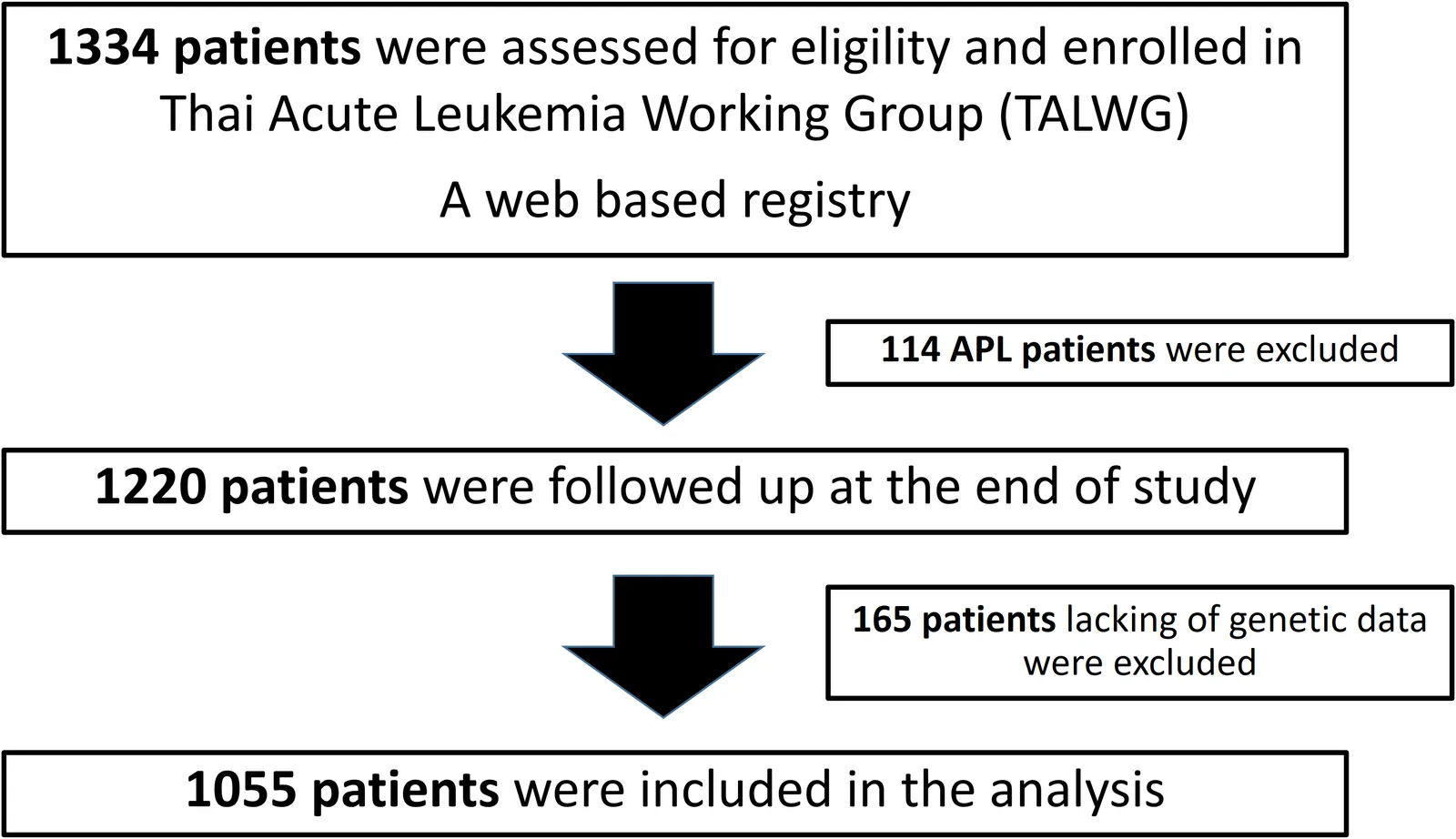
Consort flow chart of participants.

Of 1,334 patients with AML eligible for the study, 114 patients with APL and 165 patients lacking genetic data were excluded (**Figure 1**). Finally, 1,055 patients were included, the baseline characteristics of whom are summarized in **Table 1**. Baseline characteristics stratified by treatment modality are provided in **Supplementary Table 1**. The median age at diagnosis was 53.5 years without a sex predilection. *De novo* AML accounted for 78.6% of the cases. Median WBC count and percentage of blasts in peripheral blood were 23,400 cells/µL and 38%, respectively. The distribution of cytogenetic and molecular abnormalities among patients with AML is provided in **Supplementary Table 2**. Most patients (69.9%) were classified as having an intermediate risk. More than 50% of the patients were treated with the 7 + 3 regimen for induction therapy.

**Table 1:** Baseline characteristics of 1,055 patients with acute myeloid leukemia.

Characteristics	Number (%) or median (IQR)
Age (years)	53.5 (41.0–63.5)
Sex (female)	555 (52.6)
Subtype of AML	
*De novo* Secondary	829 (78.6) 226 (21.4)
ECOG	
0	43 (4.1)
1	604 (57.3)
2	268 (25.4)
3	120 (11.4)
4	20 (1.9)
Organ involvement	
Liver	127 (12.0)
Spleen	114 (10.8)
Lymph node	105 (10.0)
Skin	26 (2.5)
Oral	87 (8.2)
Testis	0 (0)
CNS	17 (1.6)
Orbit	7 (0.7)
Lung	5 (0.5)
Myeloid sarcoma	25 (2.4)
Tumor lysis syndrome	22 (2.1)
Leukostasis	90 (8.5)
Disseminated intravascular coagulation	18 (1.7)
Complete blood count	
White blood cell (cells/µL)	23,400 (4,930–67,000)
Hemoglobin (g/dL)	7.9 (6.8–9.2)
Platelet (cells/µL)	46,000 (23,000–88,000)
Blast (%)	38.0 (7.0–74.0)
Bone marrow	
Blast (%)	78.0 (45.0–90.0)
Genetic risk	
Favorable	104 (9.9)
Intermediate	737 (69.9)
Adverse	214 (20.3)
Treatment	
7 + 3 regimen	604 (57.3)
Less than 7 + 3 regimen	75 (7.1)
Hypomethylating agents	86 (8.2)
Palliative treatment	290 (27.5)

AML, acute myeloid leukemia; CNS, central nervous system; ECOG, Eastern Cooperative Oncology Group Performance Status; IQR, interquartile range.

### 3.2 Survival outcomes

**Figure 2:**
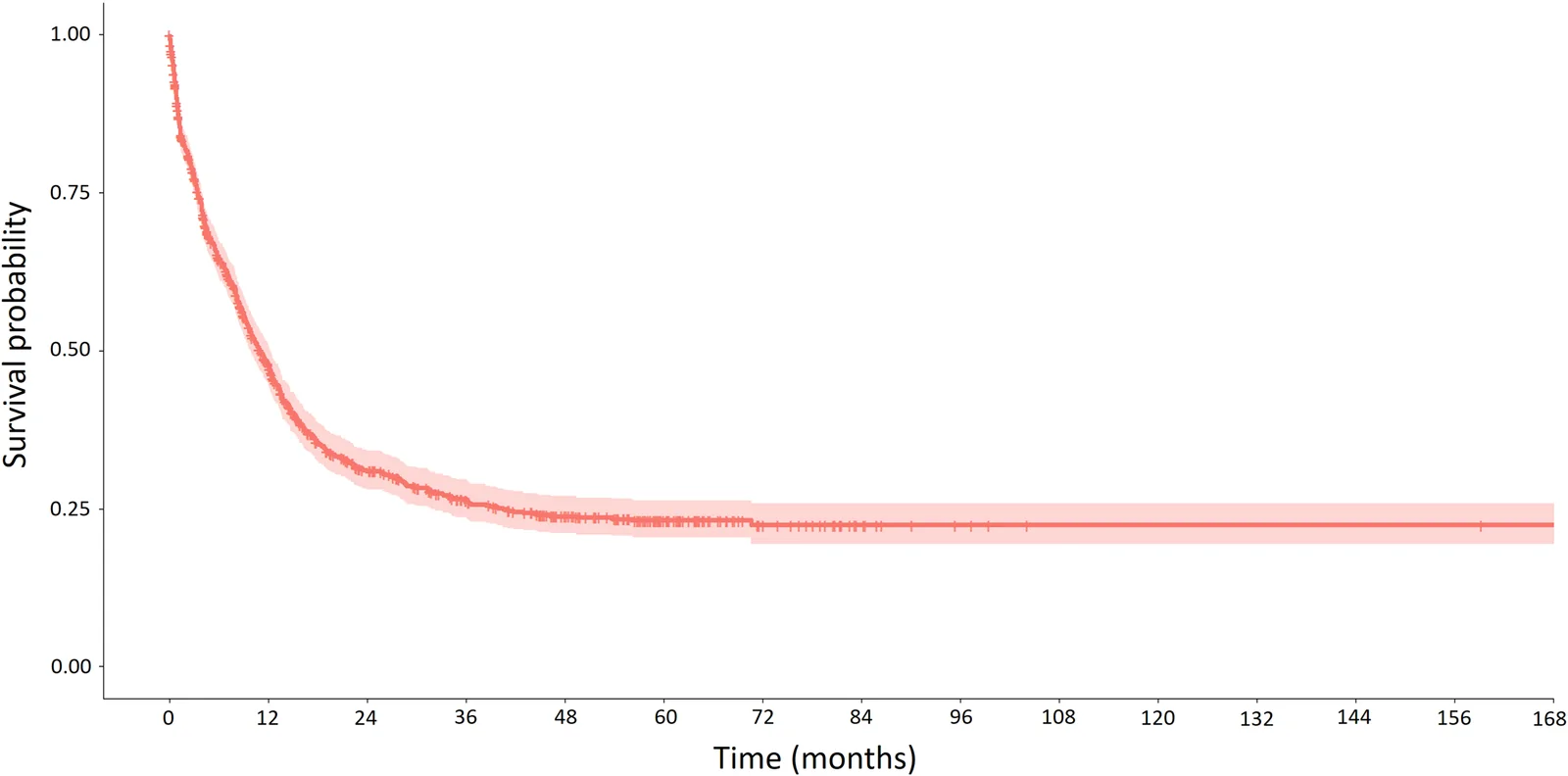
Overall survival of 1,055 patients with acute myeloid leukemia.

The median follow-up period for the entire cohort was 8.9 months (IQR 2.9–10.5). Seven hundred and fourteen patients (67.7%) had died by the time of data cutoff. The median overall survival was 10.9 months (IQR 9.8–12.2), while the 5-year and 10-year overall survival rates were 23.2% and 22.4%, respectively (**Figure 2**).

### 3.3 Prediction factors for mortality

In the univariable analysis, age, AML subtype, Eastern Cooperative Oncology Group performance status (ECOG), liver involvement, tumor lysis syndrome (TLS), leukostasis, disseminated intravascular coagulation (DIC), WBC count, percentage of blasts in peripheral blood, percentage of blasts in bone marrow, genetic risk group, and type of treatment were associated with an increased risk of death in patients with AML (**Table 2**). Multivariable analysis identified age, ECOG performance status, TLS, leukostasis, DIC, WBC count, genetic risk, and treatment type as independent predictors of mortality (**Table 2**). DIC was the strongest predictive factor of death in these patients (adjusted hazard ratio [HR] 3.13, 95% confidence interval [CI] 1.87–5.22, P < 0.001). The results of the sensitivity analysis, in which continuous predictors were retained in their original scale rather than categories, are presented in **Supplementary Table 3**.

**Table 2:** Univariable and multivariable analyses of Cox proportional hazards regression for risk of death in patients with acute myeloid leukemia.

Variables	Univariable analysis			Multivariable analysis		
	HR	95%CI	P-value	HR	95%CI	P-value
Sex (female)	1.00	0.86–1.16	0.984			
Age (>55 years)	2.15	1.85–2.50	<0.001	1.31	1.07–1.59	0.008
Subtype of AML (secondary AML)	1.74	1.47–2.06	<0.001	1.15	0.94–1.39	0.173
ECOG (>2)	2.20	1.90–2.56	<0.001	1.62	1.37–1.91	<0.001
Organ involvement						
Liver	1.34	1.08–1.66	0.009	1.23	0.98–1.56	0.070
Spleen	1.21	0.96–1.53	0.102			
Lymph node	0.90	0.70–1.16	0.418			
Skin	1.12	0.69–1.82	0.637			
Oral	0.93	0.71–1.22	0.613			
Testis	NA	NA	NA			
CNS	1.09	0.62–1.93	0.767			
Orbit	0.35	0.09–1.41	0.139			
Lung	1.52	0.57–4.07	0.401			
Myeloid sarcoma	1.52	0.99–2.35	0.058			
Tumor lysis syndrome	1.80	1.13–2.87	0.014	1.78	1.09–2.92	0.022
Leukostasis	1.50	1.17–1.92	0.001	1.34	0.99–1.81	0.062
Disseminated intravascular coagulation	3.29	2.00–5.39	<0.001	3.13	1.87–5.22	<0.001
Complete blood count						
White blood cell (>100,000 cells/µL)	1.39	1.15–1.69	<0.001	1.49	1.17–1.89	0.001
Hemoglobin (>9.0 g/dL)	0.86	0.70–1.05	0.147			
Platelet (<50 × 10^3^/µL)	1.11	0.96–1.28	0.171			
Peripheral blood blast (>30%)	0.80	0.69–0.93	0.003	1.04	0.87–1.25	0.657
Blast in bone marrow (>40%)	0.75	0.63–0.90	0.002	0.88	0.72–1.09	0.242
Genetic risk: Favorable						
Intermediate	2.18	1.63–2.93	<0.001	1.59	1.17–2.15	0.003
Adverse	2.81	2.04–3.87	<0.001	2.05	1.47–2.87	<0.001
Treatment: Palliative treatment						
7 + 3 regimen	0.22	0.18–0.26	<0.001	0.33	0.26–0.42	<0.001
Less than 7 + 3 regimen	0.39	0.29–0.52	<0.001	0.45	0.33–0.62	<0.001
Hypomethylating agent	0.39	0.30–0.52	<0.001	0.46	0.35–0.62	<0.001

AML, acute myeloid leukemia; CI, confidence interval; CNS, central nervous system; ECOG, Eastern Cooperative Oncology Group performance status; HR, hazard rati.o

### 3.4 The survival outcome prediction model

**Figure 3:**
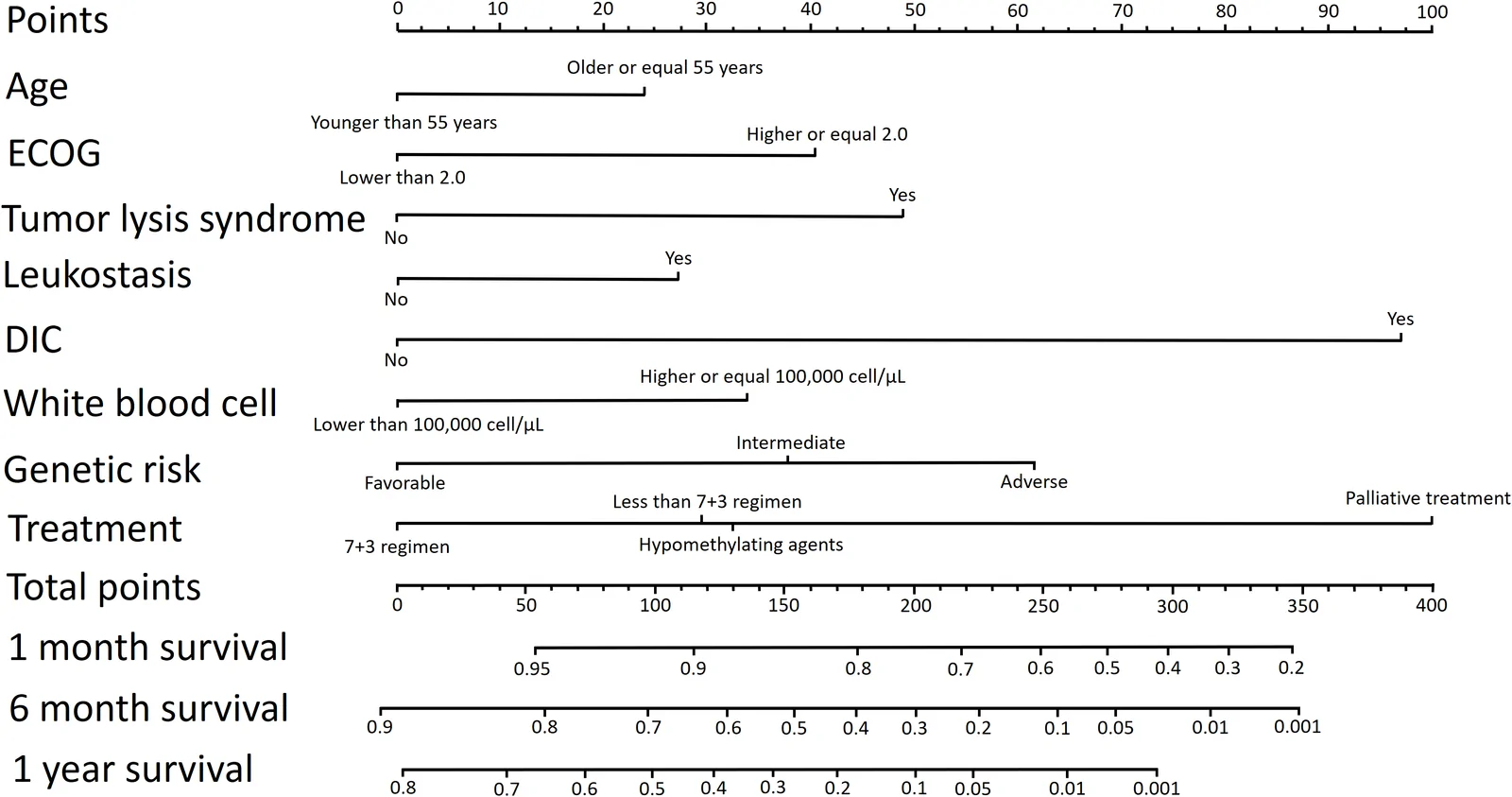
Prognostic nomogram to predict survival in patients with acute myeloid leukemia. The prediction model consists of age, ECOG performance status, tumor lysis syndrome, leukostasis, disseminated intravascular coagulation, white blood cell count, genetic risk group, and treatment of AML. ECOG; Eastern Cooperative Oncology Group performance status, DIC; disseminated intravascular coagulation.

**Figure 4:**
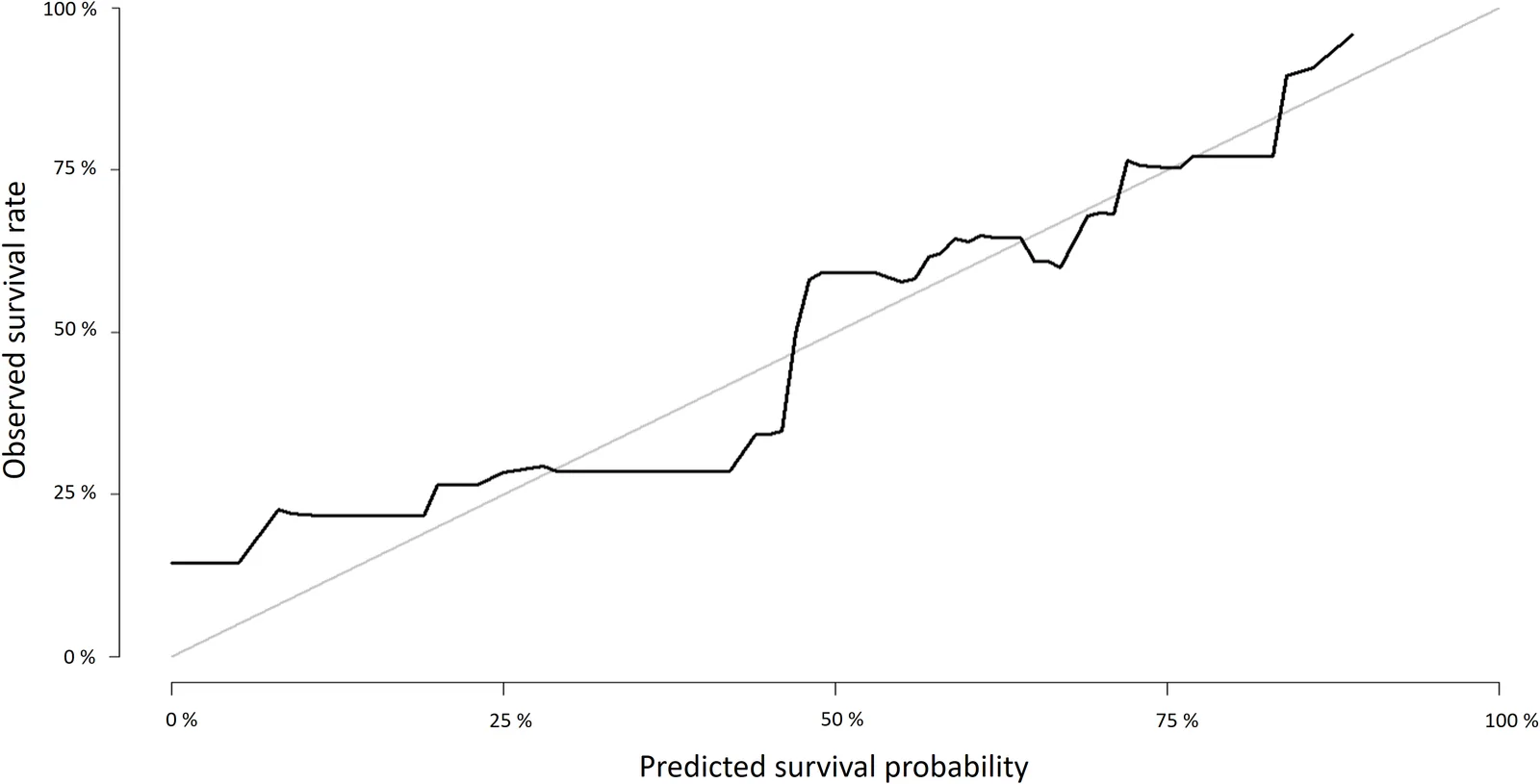
Calibration plot of survival probabilities at 6 months in 1,055 patients with acute myeloid leukemia.

After backward stepwise regression, we generated the best model with the lowest AIC, which included age, ECOG performance status, TLS, leukostasis, DIC, WBC count, genetic risk, and treatment type. A nomogram of the prediction model used to predict the probability of survival in the 1-month, 6-month, and 1-year periods is shown in **Figure 3**. The THAI-LEDGE (TLS, Hyperleukocytosis, Age, Initial treatment, Leukostasis, ECOG, DIC, and GEnetic risk) model demonstrated good discrimination (C-index = 0.743, 95% CI: 0.731–0.755) and calibration (calibration slope = 1.0) (**Figure 4**). A sensitivity analysis was performed by developing the prediction model using LASSO-penalized Cox regression, and the results are presented in **Supplementary Table 4**.

### 3.5 Internal validation of the prediction model

After bootstrapping with 500 resamples, the model’s performance remained good in the internal validation. The C-index was 0.737 (95% CI: 0.726–0.748), and the calibration plot showed good calibration of the model performance (calibration slope = 1.0) (**Supplementary Figure 1**).

## 4 Discussion

In this study, we evaluated the predictive factors for death in patients with non-APL AML and generated a model to predict survival outcomes. The median overall survival of the patients in the current study was 10.9 months. Age, ECOG PS, TLS, leukostasis, DIC, WBC count, genetic risk, and type of treatment were associated with an increased risk of death in these patients. We generated a model, namely the THAI-LEDGE model, with good performance in predicting survival in patients with AML, which was robust following bootstrap internal validation.

The prognosis of patients with AML, especially those who are ineligible for AlloSCT, remains poor (16). The 5-year overall survival rate in this study was 23.2%, comparable to that of a previous study (6). Relapse is the main cause of poor prognosis in these patients. Patients with relapsed AML generally present within 3 years of diagnosis (1). The clinical outcome of relapsed disease is poor, with a 3-year overall survival rate of <10% (17). Cooperative efforts to decrease the relapse rate of AML will improve survival outcomes in these patients.

Despite a rare occurrence, the strongest predictive factor in this study was DIC (HR 3.13, 95% CI 1.87–5.22). DIC is a life-threatening condition in patients with AML. DIC activates the coagulation system and generates intravascular thrombin and fibrin, leading to thrombus formation in small- to medium-sized vessels, consumptive coagulopathy, and life-threatening bleeding (18). DIC carries a significant thrombotic risk and poor survival outcomes in patients with AML, especially in older patients (18–20). The cornerstone of DIC treatment is the treatment of the underlying disease. Early recognition and treatment of AML can reduce DIC complications and increase the survival of these patients.

Age is a major predictive factor of AML. Many studies have demonstrated a poor prognosis in advanced-age AML (7, 8, 21). Older patients have limited tolerability to intensive chemotherapy and AlloSCT, which are the standards of care for decreasing the relapse rate in patients with AML. Multiple comorbidities, poor performance status, and vulnerability to infectious diseases are major limiting factors in these patient groups. Moreover, advanced age is associated with poor genetic risk factors, resulting in a lower probability of remission and a higher risk of relapse (22, 23). Performance status evaluation (e.g., ECOG) was incorporated into the baseline evaluation prior to treatment initiation in patients with AML. Poor ECOG performance status (ECOG > 2) was associated with poor survival in the current study. This result was compatible with that of a previous study that reported an association between poor performance status, a low CR rate after induction chemotherapy, and increased mortality (24).

Approximately 16% of patients in this study had a WBC count of more than 100,000 cells/µL, similar to a range of 5–20% reported in previous studies (25, 26). Hyperleukocytosis is associated with increased early mortality and high rates of leukostasis, DIC, and TLS (27, 28). However, only half of the patients with hyperleukocytosis developed leukostasis (symptomatic hyperleukocytosis syndrome), which occurred in approximately 9% of all patients (29). Early initiation of intensive chemotherapy is recommended to improve survival in these patients. TLS is an emergency and is considered a serious complication of AML that results from the rapid destruction of immature myeloid cells, either spontaneously or after chemotherapy. TLS is characterized by electrolyte abnormalities, acute renal dysfunction, and cardiac arrhythmias (30). TLS is associated with increased mortality during induction therapy for AML (31). This study showed that TLS was associated with poor survival outcomes (HR 1.78, 95% CI 1.09–2.92). The effective prevention, early detection, and treatment of TLS can reduce the incidence of complications and improve patient outcomes.

Our predictive model was derived from real-world data obtained from a multicenter registry. Here, we present a nomogram of the THAI-LEDGE model that is easy to apply in clinical practice. The ranges of the predicted 1-month, 6-month, and 1-year survival probabilities in this model were 20–95%, 0.1–90%, and 0.1–80%, respectively. This model had good performance, including discrimination (C-index = 0.743), with good calibration plot. Internal validation using with the bootstrapping method demonstrated consistently stable performance of the model. We have performed a LASSO-penalized Cox regression as a sensitivity analysis. The LASSO-derived model retained a larger number of predictors than the original backward stepwise AIC model. However, its predictive performance, including discrimination and calibration, was essentially comparable to that of the THAI-LEDGE model. Considering the comparable performance and the greater parsimony of the original model, we believe that retaining the THAI-LEDGE model as the final prediction model is appropriate.

DIC has been consistently associated with poor prognosis in patients with AML. Paterno et al. reported that AML patients with overt DIC had a markedly higher 30-day mortality rate than those without DIC (42.5% vs. 8.0%), while the 6-year overall survival was significantly lower (7.2% vs. 20.3%) (32). These findings suggest that DIC reflects both an aggressive disease phenotype and severe coagulation disturbances, contributing to an increased risk of mortality in patients with AML. DIC was identified as one of the predictors in our prediction model. However, only 18 patients (1.7%) in our cohort had DIC. Therefore, the estimated coefficient for this predictor may be less stable and more susceptible to sampling variability, limiting the precision of its estimated effect. Further external validation in larger cohorts, particularly those including a greater number of patients with DIC, is warranted to confirm the reproducibility and robustness of this predictor.

This study has some limitations. First, each institutional protocol regarding induction and supportive therapies may differ and may not be fully captured in the registry. Second, bone marrow analysis was not performed in 33 of the 165 patients who were excluded from the study, which resulted in an unclassifiable risk. These excluded patients may have been too sick or at a high risk of undergoing a bone marrow procedure. Therefore, they may have a very poor prognosis and could not be accounted for in our prediction model. Third, no patient with intermediate or adverse risk in our study could undergo AlloSCT because of the unavailability of stem cell donors and limited transplant center resources. As a result, these patients may experience poorer survival outcomes than those treated in a resource-rich setting. Fourth, the rate of hypomethylating agent use was only 8.2% in this study; thus, the predictive performance of this treatment may not be highly accurate. Nevertheless, the THAI-LEDGE model was developed using real-world data across diverse healthcare settings in Thailand, ensuring its generalizability to our population. Further studies, including external validation studies with more patients treated with hypomethylating agents, should be performed to validate the model’s performance. Fifth, treatment allocation was based on physician’s clinical judgement rather than random assignment and was influenced by patient’s age, performance status, comorbidities, and disease characteristics. Consequently, the inclusion of treatment type in the prediction model may introduce confounding by indication, as these factors may have influenced both treatment selection and survival outcomes. Therefore, the estimated association between treatment type and overall survival should be interpreted with caution. Finally, although backward stepwise regression with AIC was used to derive the final model, this approach has recognized limitations, including instability in predictor selection and a potential risk of overfitting. While the THAI-LEDGE model demonstrated good performance with minimal optimism following bootstrap internal validation, internal validation alone cannot establish its generalizability to independent patient populations. External validation in independent cohorts with diverse genetic backgrounds, treatment strategies, and healthcare systems is essential to confirm the model’s reproducibility, generalizability and broad clinical applicability.

## 5 Conclusion

The THAI-LEDGE model is an effective and practical tool for predicting survival in Thai patients with AML. Age, ECOG, tumor lysis syndrome, leukostasis, disseminated intravascular coagulation, WBC count, genetic risk group, and type of treatment were associated with an increased risk of death in these patients. This model is a good predictor of survival in patients with AML. However, external validation across independent cohorts with diverse patient populations, treatment strategies, and healthcare systems is necessary in further studies to confirm the generalizability of the model before broad clinical implementation.

## Data Availability

The datasets used and/or analyzed during the current study are available from the corresponding author on reasonable request.
