# Supplementary material for: Prediction model for survival outcome in patients with acute myeloid leukemia: a multicenter prospective cohort study from Thai Acute Leukemia Working Group
Source: Front Oncol. 2026 Aug 26;16:1901725. doi: 10.3389/fonc.2026.1901725 (PMC13553450; doi:10.3389/fonc.2026.1901725)
Supplement: Supplementary file 1 [file Table1.docx]

**Supplementary material**

**Fig. S1 Calibration plot of survival probabilities at 6 months using the bootstrapping method with 500 resamples for internal validation in patients with acute myeloid leukemia.**

**Table S1.** **Baseline characteristics of 1,055 patients with acute myeloid leukemia** **according to type of treatment**

| **Characteristics** | **Patients treated with 7+3 regimen**  **(n = 604)** | **Patients treated with less than 7+3 regimen**  **(n = 75)** | **Patients treated with hypomethyl-ating agents**  **(n = 86)** | **Patients treated with palliative treatment**  **(n = 290)** |
| --- | --- | --- | --- | --- |
| **Age (years), median(IQR)** | 45.9  (34.1–53.4) | 57.5  (50.6–63.6) | 66.9  (62.4–70.9) | 65.7  (57.9–73.2) |
| **Sex (female)** | 338 (56.0) | 38 (50.7) | 38 (44.2) | 141 (48.6) |
| **Subtype of AML** |  |  |  |  |
| **De novo**  **Secondary** | 528 (87.4)  76 (12.6) | 58 (77.3)  17 (22.7) | 46 (53.5)  40 (46.5) | 197 (67.9)  93 (32.1) |
| **ECOG** |  |  |  |  |
| **0** | 30 (5.0) | 1 (1.3) | 4 (4.7) | 8 (2.8) |
| **1** | 428 (70.9) | 39 (52.0) | 52 (60.5) | 85 (29.3) |
| **2** | 120 (19.9) | 21 (28.0) | 26 (30.2) | 101 (34.8) |
| **3** | 23 (3.8) | 13 (17.3) | 4 (4.7) | 80 (27.6) |
| **4** | 3 (0.5) | 1 (1.3) | 0 (0) | 16 (5.5) |
| **Organ involvement** |  |  |  |  |
| **Liver** | 75 (12.4) | 14 (18.7) | 1 (1.2) | 37 (12.8) |
| **Spleen** | 75 (12.4) | 9 (12.0) | 2 (2.3) | 28 (9.7) |
| **Lymph node** | 73 (12.1) | 9 (12.0) | 3 (3.5) | 20 (6.9) |
| **Skin** | 19 (3.1) | 1 (1.3) | 0 (0) | 6 (2.1) |
| **Oral** | 69 (11.4) | 6 (8.0) | 3 (3.5) | 9 (3.1) |
| **Testis** | 0 (0) | 0 (0) | 0 (0) | 0 (0) |
| **CNS** | 12 (2.0) | 2 (2.7) | 0 (0) | 3 (1.0) |
| **Orbit** | 5 (0.8) | 1 (1.3) | 1 (1.2) | 0 (0) |
| **Lung** | 4 (0.7) | 1 (1.3) | 0 (0) | 0 (0) |
| **Myeloid sarcoma** | 15 (2.5) | 2 (2.7) | 0 (0) | 8 (2.8) |
| **Tumor lysis syndrome** | 12 (2.0) | 1 (1.3) | 1 (1.2) | 8 (2.8) |
| **Leukostasis** | 57 (9.4) | 9 (12.0) | 1 (1.2) | 23 (7.9) |
| **Disseminated intravascular coagulation** | 7 (1.2) | 0 (0) | 0 (0) | 11 (3.8) |
| **Complete blood count** |  |  |  |  |
| **White blood cell (cells/µL), median(IQR)** | 28,550  (6,740–72,050) | 36,000  (6,875–100,250) | 8,855  (2,938–37,575) | 13,715 (3,692–57,373) |
| **Hemoglobin (g/dL), median(IQR)** | 7.9 (6.8–9.3) | 7.7 (6.8–9.5) | 7.7 (6.7–8.6) | 8.0 (6.8–9.3) |
| **Platelet count (cells/µL), median(IQR)** | 45,000 (23,000–87,000) | 41,000 (20,000–71,000) | 57,500 (27,000–133,250) | 50,000 (23,000–87,000) |
| **Blast (%), median(IQR)** | 50.0  (16.0–79.0) | 44.0  (5.0–80.5) | 16.5  (2.0–35.0) | 20.0  (2.0–65.0) |
| **Bone marrow** |  |  |  |  |
| **Blast (%),**  **median(IQR)** | 80.0  (59.7–90.0) | 80.0  (60.0–90.0) | 42.5  (26.1–79.7) | 65.1  (35.0–85.44) |
| **Genetic risk** |  |  |  |  |
| **Favorable** | 88 (14.6) | 4 (5.3) | 5 (5.8) | 7 (2.4) |
| **Intermediate** | 402 (66.6) | 53 (70.7) | 62 (72.1) | 220 (75.9) |
| **Adverse** | 114 (18.9) | 18 (24.0) | 19 (22.1) | 63 (21.7) |

AML, acute myeloid leukemia; CNS, central nervous system; ECOG, Eastern Cooperative Oncology Group Performance Status; IQR, interquartile range

**Table S2. Frequency of cytogenetic or molecular abnormalities in 1,055 patients with acute myeloid leukemia**

| **Cytogenetic or molecular study of AML** | **Number (%)** |
| --- | --- |
| t(8;21)(q22;q22); RUNX1-RUNX1T1 | 55 (5.2) |
| inv(16)(p13.1;q22) or t(16;16)(p13.1;q22); CBFB-MYH11 | 14 (1.3) |
| Mutated NPM1 without FLT3-ITD | 34 (3.2) |
| bZIP in-frame mutated CEBPA | 8 (0.8) |
| Mutated NPM1 with FLT3-ITD (without adverse-risk genetic lesion) | 30 (2.8) |
| Wild-type NPM1 without FLT-ITD | 77 (7.3) |
| t(9;11)(p21.3;q23.3); MLLT3-KMT2A | 6 (0.6) |
| t(6;9)(p23;q34.1); DEK-NUP214 | 5 (0.5) |
| t(v;11q23.3); KMT2A rearranged | 23 (2.2) |
| t(9;22)(q34.1;q11.2); BCR-ABL1 | 14 (1.3) |
| inv(3)(q21.3;q26.2) or t(3;3)(q21.3;q26.2); GATA2, MECOM(EVI1) | 4 (0.4) |
| -5 or del(5q); -7; -17/abn(17p) | 76 (7.2) |
| Complex karyotype | 114 (10.8) |
| Monosomal karyotype | 50 (4.7) |
| Mutated RUNX | 10 (0.9) |
| Mutated ASXL1 | 5 (0.5) |
| Mutated TP53 | 9 (0.9) |

**Table S3.** **Univariable and multivariable analyses of Cox proportional hazards regression for risk of death in patients with acute myeloid leukemia (with continuous data)**

| **Variables** | **Univariable analysis** | | | **Multivariable analysis** | | |
| --- | --- | --- | --- | --- | --- | --- |
|  | HR | 95% CI | P-value | HR | 95% CI | P-value |
| **Sex (female)** | 1.00 | 0.86–1.16 | 0.984 |  |  |  |
| **Age** | 1.03 | 1.02–1.03 | <0.001 | 1.01 | 1.00–1.01 | 0.006 |
| **Subtype of AML (secondary AML)** | 1.74 | 1.47–2.06 | <0.001 | 1.16 | 0.96–1.41 | 0.131 |
| **ECOG** | 1.77 | 1.62–1.93 | <0.001 | 1.36 | 1.23–1.51 | <0.001 |
| **Organ involvement** |  |  |  |  |  |  |
| **Liver** | 1.34 | 1.08–1.66 | 0.009 | 1.24 | 0.99–1.56 | 0.065 |
| **Spleen** | 1.21 | 0.96–1.53 | 0.102 |  |  |  |
| **Lymph node** | 0.90 | 0.70–1.16 | 0.418 |  |  |  |
| **Skin** | 1.12 | 0.69–1.82 | 0.637 |  |  |  |
| **Oral** | 0.93 | 0.71–1.22 | 0.613 |  |  |  |
| **Testis** | NA | NA | NA |  |  |  |
| **CNS** | 1.09 | 0.62–1.93 | 0.767 |  |  |  |
| **Orbit** | 0.35 | 0.09–1.41 | 0.139 |  |  |  |
| **Lung** | 1.52 | 0.57–4.07 | 0.401 |  |  |  |
| **Myeloid sarcoma** | 1.52 | 0.99–2.35 | 0.058 |  |  |  |
| **Tumor lysis syndrome** | 1.80 | 1.13–2.87 | 0.014 | 1.74 | 1.06–2.86 | 0.028 |
| **Leukostasis** | 1.50 | 1.17–1.92 | 0.001 | 1.38 | 1.01–1.87 | 0.041 |
| **Disseminated intravascular coagulation** | 3.29 | 2.00–5.39 | <0.001 | 2.99 | 1.79–4.98 | <0.001 |
| **Complete blood count** |  |  |  |  |  |  |
| **White blood cell** | 1.00 | 1.00–1.00 | 0.008 | 1.00 | 1.00–1.00 | 0.003 |
| **Hemoglobin** | 0.96 | 0.92–0.99 | 0.017 | 0.98 | 0.94–1.02 | 0.251 |
| **Platelet** | 1.00 | 1.00–1.00 | 0.362 |  |  |  |
| **Peripheral blood blast** | 1.00 | 1.00–1.00 | 0.178 |  |  |  |
| **Blast in bone marrow** | 1.00 | 1.00–1.00 | 0.01 | 1.00 | 1.00–1.00 | 0.400 |
| **Genetic risk: Favorable** |  |  |  |  |  |  |
| **Intermediate** | 2.18 | 1.63–2.93 | <0.001 | 1.50 | 1.11–2.03 | 0.008 |
| **Adverse** | 2.81 | 2.04–3.87 | <0.001 | 1.98 | 1.42–2.77 | <0.001 |
| **Treatment: Palliative treatment** |  |  |  |  |  |  |
| **7+3 regimen** | 0.22 | 0.18–0.26 | <0.001 | 1.36 | 1.02–1.83 | 0.039 |
| **Less than 7+3 regimen** | 0.39 | 0.29–0.52 | <0.001 | 1.42 | 1.04–1.93 | 0.025 |
| **Hypomethylating agent** | 0.39 | 0.30–0.52 | <0.001 | 2.90 | 2.27–3.69 | <0.001 |

AML, acute myeloid leukemia; CI, confidence interval; CNS, central nervous system; ECOG, Eastern Cooperative Oncology Group performance status; HR, hazard ratio

**Table S4.** **Prediction model for risk of death in patients with acute myeloid leukemia using LASSO-penalized Cox proportional hazards regression**

| **Variables** | HR | 95% CI | P-value |
| --- | --- | --- | --- |
| **Sex (female)** | 1.12 | 0.96–1.30 | 0.142 |
| **Age (>55 years)** | 0.76 | 0.62–0.93 | 0.006 |
| **Subtype of AML (secondary AML)** | 1.18 | 0.98–1.42 | 0.081 |
| **ECOG (>2)** | 0.64 | 0.54–0.75 | <0.001 |
| **Organ involvement** |  |  |  |
| **Liver** | 1.15 | 0.88–1.51 | 0.300 |
| **Spleen** | 1.17 | 0.88–1.55 | 0.287 |
| **Oral** | 0.39 | 0.09–1.57 | 0.182 |
| **Myeloid sarcoma** | 1.66 | 1.06–2.60 | 0.027 |
| **Tumor lysis syndrome** | 1.57 | 0.96–1.57 | 0.072 |
| **Leukostasis** | 1.42 | 1.05–1.92 | 0.022 |
| **Disseminated intravascular coagulation** | 2.92 | 1.74–4.88 | <0.001 |
| **Complete blood count** |  |  |  |
| **White blood cell (>100,000 cells/µL)** | 0.66 | 0.52–0.83 | <0.001 |
| **Hemoglobin (>9.0 g/dL)** | 1.16 | 0.94–1.43 | 0.171 |
| **Blast in bone marrow (>40%)** | 1.18 | 0.97–1.42 | 0.100 |
| **Treatment: Palliative treatment** |  |  |  |
| **7+3 regimen** | 1.48 | 1.10–1.99 | 0.009 |
| **Less than 7+3 regimen** | 1.49 | 1.09–2.03 | 0.012 |
| **Hypomethylating agent** | 3.33 | 2.63–4.20 | <0.001 |

The C-index was 0.740 (95% CI: 0.727-0752). The calibration plot showed good calibration with calibration slope = 1.

AML, acute myeloid leukemia; CI, confidence interval; ECOG, Eastern Cooperative Oncology Group performance status; HR, hazard ratio
